# Hair follicle immune privilege in autoimmune and immune-mediated alopecias: paths toward reestablishing immune tolerance

**DOI:** 10.3389/fmed.2026.1805170

**Published:** 2026-03-31

**Authors:** Pedram Shafiei-Jahani, Xin Li, Amitis Akbari, Emily Haniff, Melissa Ziprick, Ryan Carlisle, Carolyn Goh, Vanessa Holland, Omid Akbari

**Affiliations:** 1 David Geffen School of Medicine at the University of California, Los Angeles, Los Angeles, CA, United States; 2 Department of Molecular Microbiology and Immunology, Keck School of Medicine, University of Southern California, Los Angeles, CA, United States; 3 Division of Dermatology, Department of Medicine, University of California, Los Angeles, Los Angeles, CA, United States

**Keywords:** hair follicle immune privilege, immune privilege collapse, alopecia areata, lichen planopilaris, frontal fibrosing alopecia, discoid lupus erythematosus, central centrifugal cicatricial alopecia, Network Engineering for Site-Specific Tolerance (NEST)

## Abstract

Autoimmune and immune-mediated alopecias demonstrate how a site-specific failure of immune privilege can produce reversible or irreversible hair loss. In alopecia areata (AA), cytotoxic injury is concentrated at the anagen bulb but preserves the stem cell pool, permitting regrowth following immune suppression. Lichen planopilaris (LPP) and frontal fibrosing alopecia (FFA) feature a spectrum of chronic interface dermatitis that destroys the stem cell niche, leading to permanent fibrosis. These entities overlap in bulge-targeted inflammation and histopathology but diverge in clinical distribution and demographic risk. Discoid lupus erythematosus (DLE) of the scalp reflects immune-complex–driven complement injury to the upper follicle, whereas central centrifugal cicatricial alopecia (CCCA) represents a fibroblast-dominant process associated with chronic stress and is sustained by stromal remodeling. Here, we frame these entities as four follicular immune network archetypes defined by the axial locus of immune privilege collapse, effector programs, loss of tolerogenic programs, stromal trajectories, and dermoscopic correlates. Using a Network Engineering for Site-Specific Tolerance (NEST) framework, we situate these archetypes within a three-tier taxonomy comprising intrinsic follicular privilege, local tolerogenic repertoires, and stromal checkpoint circuits. Specifically, AA localizes to the anagen bulb, DLE spans the upper follicle and interfollicular epidermis, LPP/FFA targets the bulge stem cell niche, and CCCA converges on the upper follicle–interfollicular epidermis unit. Within their respective niches, AA and DLE exhibit comparatively resolved clonotypic effector modules dominated by pathogenic T cells or B cells, whereas LPP/FFA and CCCA are framed as polyclonal networks in which the failure of tolerogenic programs or stress signaling pathways constitutes the dominant archetypal engine.

## Introduction: NEST taxonomy anchored in immune tolerance

**Figure 1:**
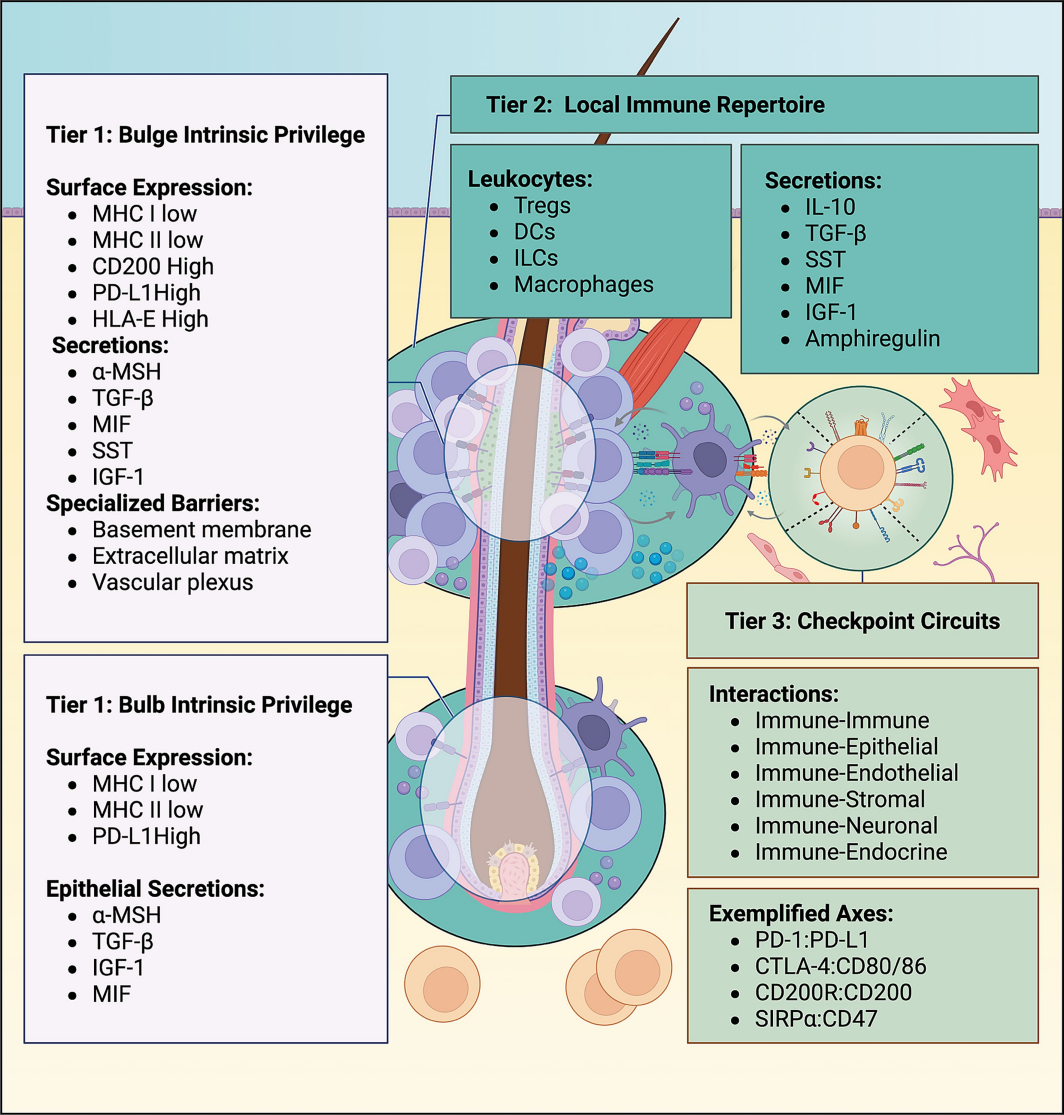
NEST taxonomy for hair follicle immune tolerance across three tiers. The Network Engineering for Site-Specific Tolerance (NEST) framework organizes follicular immune privilege into three distinct tiers: (1) Intrinsic epithelial and stromal privilege programs, (2) local tolerogenic immune repertoires, and (3) checkpoint circuits that restrain inflammatory escalation.

Within the Network Engineering for Site-Specific Tolerance (NEST) framework (Figure 1), the intrinsic privilege tier lies within the follicular epithelium and adjacent stroma. The bulb corresponds to the anagen matrix and lower follicle, the bulge to the epithelial stem-cell niche at the isthmus, and the upper follicle to the infundibuloisthmic segment above the bulge (1). Immune privilege (IP) is moderate at the bulge and strongest at the bulb, primarily due to additional regulatory programs and checkpoints (1, 2). In both regions, keratinocytes downregulate MHC class I/II during homeostasis and deploy various tolerogenic networks to suppress antigen presentation and danger signaling within their respective niches (2, 3). Additional structural barriers, including a specialized vascular plexus, work in concert with local tolerogenic programs to ensure quiescence (2–4).

The second tier encompasses the immediate perifollicular microenvironment, particularly tissue-resident immune cells such as regulatory T cells (Tregs), tolerogenic dendritic cells (tDCs), and likely innate lymphoid cell (ILC) subsets (5–13). Tregs cluster around follicles and protect stem-cell compartments in genetic and spatial disruption models; their deficiency induces alopecia across multiple model systems (14, 15). tDCs play a complementary role by presenting antigens in a non-inflammatory context, secreting IL-10 and TGF-*β* to support Treg function. Furthermore, emerging ILC subsets can act as innate regulatory hubs in various tissue barriers outside of alopecia by supplying IL-2 and IL-10, engaging amphiregulin-dependent repair pathways, and expressing ICOSL and OX40L for contact-dependent interactions with resident Tregs (5, 6, 9, 16). Although ILCs have not been comprehensively mapped in autoimmune alopecias, their ability to sustain Treg-mediated tolerance at barrier sites warrants additional studies in this context.

The third tolerance tier is constituted by a broad set of checkpoint circuits, exemplified by PD-1, CTLA-4, VISTA, SIRPα, and CD200R (17–25). These inhibitory axes can result in immune–immune and epithelial–immune tolerogenic interactions. For instance, the CD200-positive PD-L1-positive bulge stem cells engage their respective receptors on local macrophages, dendritic cells, mast cells, and innate lymphoid cells (17–19, 26–28). Similarly, PSGL-1, which is broadly expressed on T cells and myeloid cells, can engage VISTA on other inflammatory myeloid cells, thereby abrogating tissue injury. Furthermore, the ubiquitously expressed CD47 ligand on keratinocytes, endothelial cells, and immune cells can engage SIRPα to suppress inflammatory antigen-presenting cells (APCs) (17). The engagement of inhibitory receptors on Tregs and innate nodes forms microdomains in which immune surveillance remains intact, but inflammatory responses are restrained (22, 29, 30). Checkpoint engagements, combined with cytokines and chemokines, license the recruitment, retention, and function of regulatory cells, thereby setting a high activation threshold for inflammatory pathways (22, 30). Genetic susceptibility, stress signals, and immunomodulatory therapies may tip the balance within this tier; therefore, additional studies in alopecia cohorts are needed to comprehensively map all regulatory pathways and create a scaffold for future immune engineering strategies (29, 30).

The three NEST tiers are defined by function rather than by mutually exclusive molecules. The first tier governs follicular antigenic visibility, intrinsic barrier integrity, and danger signaling; the second tier reflects the composition and spatial organization of the surrounding local immune repertoire, which can either preserve tolerance or permit pathogenic barrier injury; and the third tier encompasses the receptor-ligand interaction networks between epithelial, stromal, and immune cells across the first two tiers, thereby setting the thresholds for immune entry and activation within the follicular microenvironment.

## Intrinsic follicular privilege across archetypes

The first axis of divergence in the NEST framework is the axial level along the bulb–bulge–upper follicle continuum where intrinsic privilege fails.

**Figure 2:**
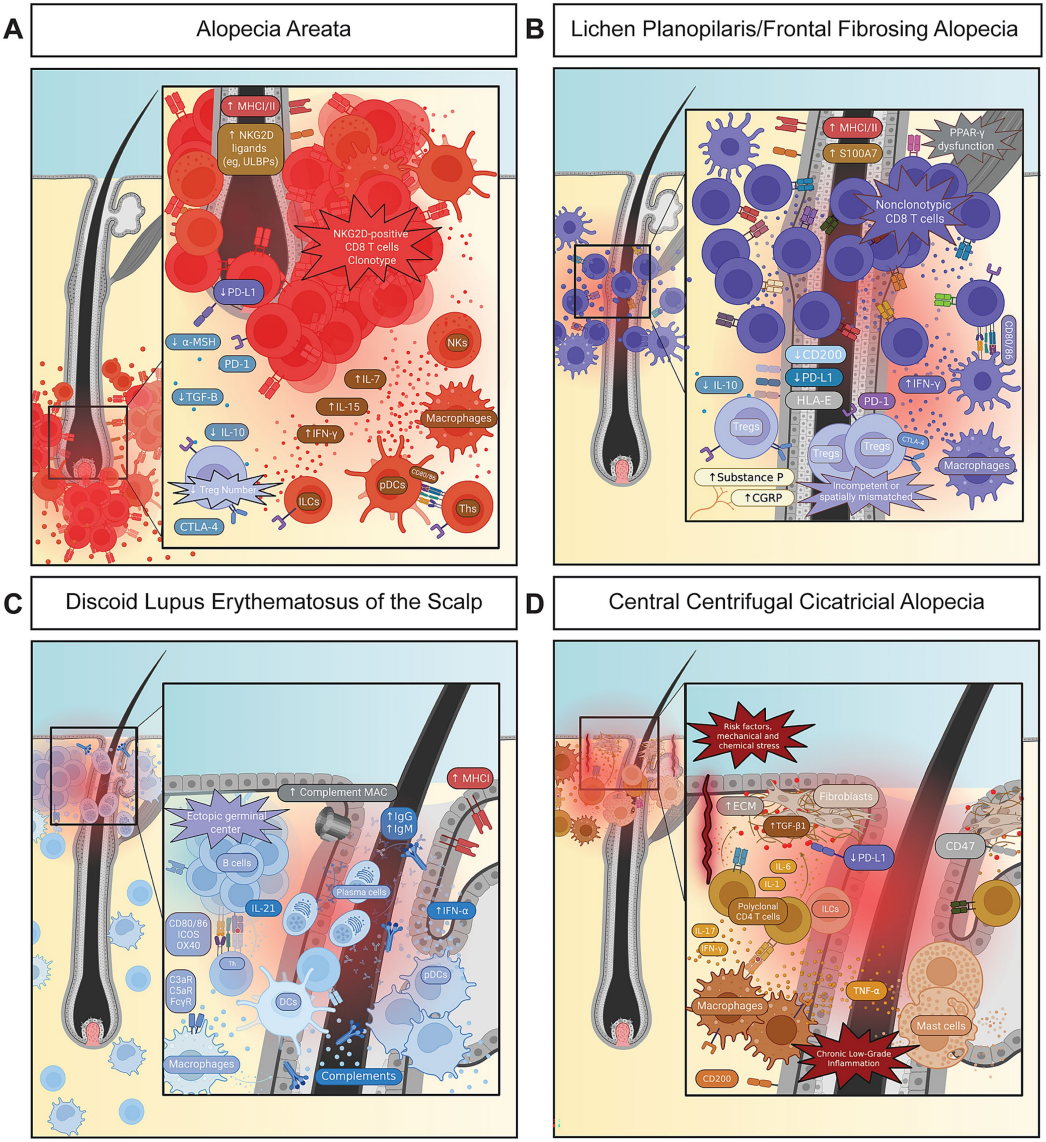
Autoimmune and immune-mediated alopecias as archetypes linked to compartment-specific failure of tolerance and downstream tissue outcomes. **(A)** Alopecia areata: bulb-restricted cytotoxic inflammation with relative preservation of the epithelial stem-cell reservoir, consistent with clinical reversibility. **(B)** Lichen planopilaris and frontal fibrosing alopecia: infundibuloisthmic- and bulge-directed interface inflammation that erodes the stem-cell niche and licenses a scarring trajectory. **(C)** Discoid lupus erythematosus of the scalp: immune-complex- and complement-dependent injury centered on the upper follicle and adjacent dermoepidermal unit. **(D)** Central centrifugal cicatricial alopecia: stress-primed, fibroblast-dominant remodeling with chronic, low-amplitude inflammation that consolidates fibrosis.

In AA, clinicopathologic observations in patients, together with mechanistic evidence from experimental models, place the dominant intrinsic dysfunction at the hair bulb, whereas systemic immune tolerance remains largely intact (31, 32). Bulbar keratinocytes downregulate PD-L1 and upregulate MHC class I/II and NKG2D ligands, such as ULBPs (Figure 2A). Moreover, *α*-MSH, TGF-*β*, IL-10, and IDO secretion can be abrogated, contributing to a diminished tolerance around the bulb and converting a low-visibility matrix into a compartment accessible to cytotoxic surveillance (30, 32, 33). The bulge stem-cell niche with additional tolerogenic programs remains relatively spared, early fibrosis is minimal, and the infiltrate forms a classic “swarm of bees” (Table 1) around the lower follicle with little isthmic interface change (31).

**Table 1:** Dermoscopic patterns and histopathologic correlates in immune-mediated alopecias.

Condition	Sex predominance	Dermoscopic features	Histological features
AA	Modest female predominance F:M ~1.3:1	Follicular openings preserved. Yellow dots and short vellus hairs relatively sensitive. Black dots and exclamation/tapered hairs more specific.	Early: peribulbar lymphocytic infiltrate (“swarm of bees”), catagen/telogen shift, trichomalacia, pigment casts. Chronic: miniaturization with nanogen hairs; inflammation often reduced.
LPP and FFA	Strong female predominance LPP: F:M ~4.9:1 FFA: F:M ~31:1	Both with perifollicular erythema with tubular perifollicular scale or casts, progressive loss of follicular ostia, and white scar-like patches. LPP with more prominent casts and milky red areas. FFA with isolated lonely terminal hairs within the frontotemporal band. Blue gray dots variably reported.	Lichenoid/interface lymphocytic inflammation targeting infundibulum/isthmus with early sebaceous gland loss and concentric perifollicular lamellar fibrosis, progressing to fibrosed follicular tracts. FFA mirrors this LPP-spectrum pattern, with early vellus involvement.
DLE of scalp	Strong female predominance Adult F:M ~2–5:1 Pediatric F:M ~1.8:1	Follicular openings reduced or absent. Follicular red dots, follicular keratotic plugs, arborizing or tortuous vessels, large yellow dots, blue/gray peppering with interfollicular extension, white structureless areas. Honeycomb disruption in darker skin phototypes.	Vacuolar interface dermatitis of epidermis and follicular epithelium with basement membrane thickening, follicular plugging, periadnexal inflammation, dermal mucin, pigment incontinence, and scarring with pilosebaceous atrophy. DIF often shows granular deposits at the dermoepidermal junction and/or follicular junction.
CCCA	Possible female predominance in patients of African ancestry	Follicular openings reduced or absent. Peripilar white gray halo, often 2 to 3 hairs per ostium. Irregular pinpoint white dots and white patches. Miniaturization and mixed shaft diameters. True casts absent.	Premature inner root sheath desquamation with eccentric outer root sheath thinning, concentric lamellar perifollicular fibroplasia with variable lymphocytic inflammation and follicular dropout. Later retained shaft fragments with granulomatous inflammation. Polytrichia may be present.

In LPP and FFA, comparative immunopathologic profiling of human lesional scalp biopsies places the inflammatory band at the infundibuloisthmic region, showing basal vacuolar changes and apoptotic keratinocytes around the bulge (34). Bulge keratinocytes downregulate PD-L1, CD200, and HLA-E and upregulate MHC class I/II, β2-microglobulin, and alarmins such as S100A7 (34, 35). In addition to the immune privilege markers mentioned for AA, the robust tolerance network of the bulge fails with abrogated expression of K15, β1-integrin, and TGF-β2 signaling, collectively denoting a destabilized stem-cell niche (Figure 2B). Moreover, microarray analysis revealed a loss of epithelial hair-follicle stem-cell signatures together with increased expression of T-cell activation and binding markers in active LPP, supporting the inflammatory destabilization of the bulge niche (34). Taken together, since these dysregulations occur close to the epithelial stem-cell pool, immune attack, stem-cell depletion, and early fibrosis become tightly coupled.

Scalp DLE and CCCA both anchor their primary intrinsic dysfunction in the upper follicle and its interface with the interfollicular epidermis, yet they erode privilege through different mechanisms (Figures 2C,D) (36–40). In scalp DLE, human histopathology and immunopathology studies suggest that keratinocytes and endothelial cells at the dermoepidermal junction experience chronic exposure to immune complexes, complement, and type I interferons (40–42). This environment drives MHC class I upregulation, expands interferon-stimulated gene programs, sustains antigen presentation, and increases immune surveillance of the upper follicle (41–43).

In CCCA, emerging evidence, observational, and genetic association studies suggest that privilege erosion may arise from recurrent mechanical or chemical injury at the infundibulum and ostium (37, 38). Microtrauma may disrupt epithelial integrity and junctional architecture, weaken barrier function, and activate stress-repair programs in upper-follicle keratinocytes (37, 38). Early clinicopathologic and transcriptomic data further suggest reduced checkpoint tone in this niche, resulting in a lowered activation threshold for bystander lymphocytes (36, 37, 39, 44). The intrinsic tier shifts from a normally quiet, partially shielded upper follicle to a chronically stressed, checkpoint-poor environment.

## Local-regulatory repertoires and effector engines

The second NEST tier comprises cellular drivers of each archetype, as well as the regulatory cell repertoires that fail to protect against autoimmunity.

In AA, patient lesional transcriptional and single-cell studies, together with mechanistic murine models, support a bulb-centered clonotypic effector program dominated by NKG2D-positive CD8 T cells (Figure 2A) (30, 45–48). These antigen-driven clonotypes often persist despite apparent clinical improvement, a finding consistent with a durable tissue-resident memory T-cell (TRM) reservoir embedded within an immunologically primed network maintained by IL-7 and IL-15 from peribulbar stromal and accessory cells (45, 46, 48, 49). In human studies, Tregs have been reported as numerically reduced around the bulb (14, 50, 51). The cytokine milieu reported has been mixed but strongly skewed toward type 1 immunity (50–52). Systemic immunity remains largely preserved, and the survival of the bulge stem cell reservoir permits regrowth once inflammatory pressure near the bulb is relieved (30, 52).

In LPP and FFA, comparative human lesional immunopathology and immunologic profiling studies suggest that Tregs are present but functionally impaired (34, 53). Paradoxically, bulk FFA tissue shows increased numbers of FOXP3-positive Treg, yet these cells fail to promote tolerance or protect bulge stem cells (Figure 2B). This suggests reduced Treg-based regulation arising from spatial mislocalization, functional exhaustion, or both (34, 53, 54). In further contrast to AA, the effector engines do not appear to be clonotypic, and IL-7 and IL-15 survival loops are less defined. Instead, current literature points to TRM persistence associated with chronic IFN-*γ* exposure, neuropeptides (e.g., substance P and CGRP), and stress circuits (44, 54–56). Though future studies may uncover evidence supporting a clonotypic T-cell response in some cases, the current evidence favors a model in which non-clonotypic CD8 T-cells, TRMs, chronic IFN-γ and neuropeptide circuits, and functionally incompetent Tregs enable autoimmunity.

In DLE, human lesional transcriptomic and immunohistologic studies suggest that the effector engine is centered on a B-cell–dominant module (41, 42, 57–60). Lesional scalp contains B-cell aggregates and plasma cells with clonal expansion, somatic hypermutation, and local antigen-driven maturation, potentially supporting ectopic germinal-center–like structures (Figure 2C) (59, 61, 62). Autoantibodies from these clones form immune complexes that activate complement and drive upper-follicle injury (41, 60, 63). Plasmacytoid dendritic cells (pDCs), through type I-interferon secretion, further condition keratinocytes and stromal cells to upregulate MHC class I, CXCR3 ligands, and adhesion molecules. This, thereby, establishes a feed-forward chemotactic and cytotoxic milieu that promotes a chronic, self-propagating pattern of epithelial and follicular damage. Furthermore, helper and cytotoxic T cells provide cognate help, reinforcing the chronic immune-complex and type I interferon milieu that sustains autoreactivity (41, 57, 60, 64).

In CCCA, clinicopathologic and emerging transcriptomic studies suggest that lesional infiltrates are relatively sparse, consisting of polyclonal CD4 T cells that contribute to sustaining low-grade inflammation via cytokine support (Figure 2D) (39, 44, 65). B-cell aggregates and immune-complex deposition are minimal (39, 66). A characteristic regulatory or TRM signature has not been defined, and regulatory cells appear to operate in the background of fibroblast-driven remodeling (44, 65). Within this setting, polyclonal T cells and innate immune cells may contribute to a low-grade, chronic inflammatory state with a strong profibrotic component.

## Checkpoint circuits

Compared with the first two tiers, mechanistic insights supporting the third NEST tier rely more heavily on broader human immunobiology and preclinical murine studies, as direct functional perturbation studies in human alopecic scalps remain of limited feasibility.

In AA, human lesional observations and immunophenotyping studies, together with mechanistic murine models, support the presence of a bulb compartment that cannot effectively suppress inflammatory APCs and clonotypic CD8 T cells (Figure 2A) (30, 32, 67, 68). PD-L1 expression is diminished in both the bulge and the bulb, potentially leaving PD-1 insufficiently engaged across these compartments, thereby lowering the threshold for immune disruption. Overt inflammation nonetheless remains concentrated at the bulb rather than the bulge, which is more effectively insulated by other checkpoints (14, 30). Concurrent production of type I interferons and IFN-*γ* by pDCs and NKG2D-positive CD8 T cells induces IL-15 and CXCR3 ligands, all of which stabilize cytotoxic NKG2D-positive TRMs (46, 52, 69). The lack of PD-L1 likely further results in reduced PD-1 engagement on NKG2D-positive TRMs, limiting their suppression, and on Tregs, weakening FOXP3 stability (30, 68, 70, 71).

Dysregulations of circuits within LPP and FFA are less well defined than in AA. The bulge’s privilege programming and tolerogenic architecture are more extensive and robust than those of the bulb; yet they fail to protect against a nonclonotypic response (Figure 2B). Under homeostasis, CD200-positive and PD-L1-positive bulge stem cells deliver parallel tolerogenic signals to adjacent immune cells; however, both of these checkpoint axes are downregulated in LPP and FFA (54, 72–78). In addition to checkpoint ligands directly expressed by the bulge epithelial cells, other checkpoint ligand–receptor axes within the surrounding immunity can contribute to tolerance within this region (79–84). For instance, CTLA-4 on Tregs controls antigen presentation by macrophages and dendritic cells via removing CD80 and CD86 through trans-endocytosis (73, 75, 76, 85, 86). In LPP/FFA, such checkpoints can be lost or outcompeted by inflammatory programs (54, 77, 87, 88). For instance, APCs chronically conditioned by IFN-*γ* can maintain high expression of stimulatory ligands, such as CD80, CD86, ICOSL, and OX40L, thereby providing polyclonal CD8 TRMs with strong co-stimulatory signals (44, 56, 88, 89). As fibrosis progresses, TGF-β1 and connective tissue growth factor (CTGF) narrow and stiffen the perifollicular sheath, likely displacing Tregs and amplifying the loss of regulation (56, 88, 90). Recent reports also suggest that neuropeptides, such as substance P and calcitonin gene-related peptide (CGRP), can contribute to this pathogenic dysregulation (55, 91). Both LPP and FFA show strong female predominance, with FFA primarily affecting postmenopausal women, together suggesting an incompletely understood role for endocrine–immune axis regulation (92, 93).

In DLE, the inflammation within the upper follicle is driven by immune complex and complement activation that can overwhelm inhibitory checkpoint pathways (94–97). The nucleic-acid–rich immune complexes activate Fcγ receptors and endosomal sensors, while the anaphylatoxins C3a/C5a amplify myeloid recruitment and interferon production (Figure 2C) (94, 96, 98–100). Activating signals saturate activating Fcγ receptors and exceed the regulatory input from inhibitory FcγRIIb, complement regulators, and checkpoints, driving APCs into a high-interferon, high-co-stimulatory state that is potentially sustained through the ICOS–ICOSL and OX40–OX40L axes (95, 97, 100). Though additional studies are needed, Tregs likely receive inadequate support, resulting in reduced IL-10 production and poor control of follicular helper T (Tfh) cell expansion (58, 101, 102).

In CCCA, emerging evidence suggests that chronic mechanical and chemical trauma may progressively shift regulatory control from follicular immune privilege toward an innate wound-healing and matrix mechanosensing program (36, 38, 103, 104). Under homeostatic conditions, CD200 on the infundibular epithelium engages CD200R on innate lymphoid cells, mast cells, or macrophages and potentially cooperates with additional inhibitory checkpoints, such as SIRPα interacting with CD47 and VISTA, which are typically expressed on these immune cells, to confine damage responses to brief, self-limited bursts of IL-1, IL-6, and TGF-*β* (Figure 2D) (75, 105, 106). With ongoing traction, heat, and chemical exposure, the expression of regulatory ligands can decline at the epithelial interface, potentially leaving their receptors insufficiently ligated (72, 75, 105, 107). With chronic low-grade inflammation, follicular and perifollicular fibroblasts adopt an αSMA-positive myofibroblast phenotype with increased deposition of collagen I, collagen III, and type VI collagen (COL6A1) and the induction of matrix metalloproteinases, while integrin-mediated mechanotransduction reinforces a TGF-β–dominant profibrotic state (65, 108–111).

## NEST dermoscopic correlates

Dermoscopy offers a high-resolution, noninvasive window into follicular architecture and associated vascular and keratin patterns, enabling clinical diagnosis, phenotyping, and longitudinal tracking in alopecias (112–114). Within the NEST framework, dermoscopic features (Table 1) in clinicopathologic studies hint at the aforementioned networks.

In AA, dermoscopy shows preserved follicular openings and prominent yellow dots, indicating an upper follicular apparatus whose structural programs remain intact, with infundibula patent but transiently unoccupied after matrix injury imposed by IL-15–supported, clonotypic CD8 circuits (31, 32, 115, 116). Dystrophic hairs, broken hairs, black dots, and exclamation mark hairs represent the surface correlates of this bulb-directed immune response (32, 115–117). IFN-*γ* and stress-ligand–driven injury constricts the proximal shaft and perturbs keratinization, producing fragile fibers that taper and fracture at or just above the ostium (52, 118). The emergence of numerous short regrowing hairs signals the renewal of the bulge-centered repair axis once type-1-skewed immunity withdraws its pressure from the lower follicle (115–117).

In LPP and FFA, dermoscopy reveals perifollicular erythema and tubular scale, rendering the autoimmune interface process as a circumferential vascular rim and a keratin sleeve that envelops the upper follicle (119–123). At this level, chemokine-positioned, nonclonotypic cytotoxic T cells likely converge with a neuropeptide-responsive circuit to form a tight, collar-like infiltrate around the ostia (36, 44, 77, 124). Progressive narrowing and eventual loss of follicular openings indicate fibroconnective remodeling that overrides and likely displaces tolerogenic programs (119, 125, 126). Perifollicular white halos demarcate the expanding stroma, consistent with the uncoupling of regulatory elements from the K15^+^ stem-cell niche (77, 122, 126). This architectural failure in FFA additionally produces the lonely hair pattern along the frontal hairline (119, 120).

In DLE, dermoscopy shows adherent scale across follicular and interfollicular skin, visualizing an immune-complex–driven interface dermatitis sustained by B-cell aggregates and type I interferon circuits (41, 127–129). Follicular plugging is seen at the infundibulum, where basement membrane disruption and abnormal desquamation produce dense keratin obstruction as inflammatory signals outcompete the inhibitory cues (41, 127, 128, 130). Arborizing or prominent telangiectatic vessels reflect chronic endothelial activation within a proangiogenic and complement-rich microenvironment (41, 131). White structureless zones and intermittent blue-gray granularity denote the terminal repair state, where collagenous scarring and pigment incontinence permanently replace the native epithelial and adnexal architecture (119, 128, 131).

In CCCA, dermoscopy initially reveals perifollicular white halos, marking the early rise of a fibroblast-centered axis that still preserves the recognizable contours of the upper follicle, characterized by sheath thickening and perifollicular collagen deposition (119, 132–134). Progressive reduction and eventual loss of follicular openings denote inflammatory programs that override tolerogenic axes and, over time, convert epithelial canals into fibrous tracts (132, 135). Broken and miniaturized hairs reflect the influence of chronic remodeling, as recurrent mechanical stress and an impaired inner root sheath support the generation of thin, fragile hair shafts (104, 136, 137). Expanding white structureless areas may represent the merger of individual fibroblast-dominated units into confluent lesions, signaling that fibrotic remodeling has fully supplanted the native immune and epithelial programs (132, 133, 135).

## Concluding remarks: the future of multi-tier therapeutics to rebuild tolerance

Within the NEST framework, durable remission requires the restoration of local tolerance, because broad immunosuppression can transiently suppress inflammation but cannot reset the local tolerogenic networks that govern long-term quiescence. The conceptual approaches outlined here are intended to illustrate how NEST can guide multi-tier regimen design rather than to define treatment protocols. In this view, immune engineering seeks to move beyond global network dampening toward tolerance restoration by repairing the dominant tolerance dysfunction in each archetype and reinforcing adjacent tiers.

In AA, the local delivery of immune privilege mediators, combined with the selective expansion of follicle-associated Tregs, for example, via topical IL-2-biased agonists or CCL22-based chemoattractants, could reinforce bulb-intrinsic and local regulatory tiers. In parallel, the rational pruning of antigen-driven clonotypic CD8 T-cell pools along the NKG2D and IL-15 axes, using IL-15 pathway antagonists or established JAK inhibitors, combined with the reinforcement of the checkpoint tier through agonism of receptors such as PD-1 or CD200R, could synergistically reestablish bulb tolerance and constrain effector persistence (48, 50, 70). In LPP and FFA, immune engineering seeks to preserve and, where possible, in adjacent follicles, reconstruct bulge-intrinsic privilege and local regulatory tiers by attenuating IFN-*γ*-driven and neurogenic injury circuits (138). Combinations of JAK-targeted inhibition of inflammatory T cells, agonistic stimulation of tolerogenic checkpoints on the local immune repertoire, neuromodulatory interventions, or strategies designed around restoring Treg function can recalibrate the milieu toward a more tolerogenic and antifibrotic state. In DLE, therapeutic strategies center on restoring B-cell tolerance and repairing checkpoints at the upper follicle and dermoepidermal junction. Strategies can combine clinically used lupus therapies, including B-cell depletion (e.g., rituximab, off-label) or modulation of B-cell survival pathways (e.g., belimumab), with type I interferon blockade (e.g., anifrolumab), as well as complement inhibition or selective targeting of nucleic acid-sensing pathways. In addition, efficacy can be supplemented by protolerogenic interventions, such as dendritic cell-based vaccines or checkpoint agonism, to re-impose B-cell tolerance and permit the re-emergence of immune privilege programs in residual follicles (60, 139, 140). In CCCA, immune engineering would primarily target fibroblast programming and checkpoints within the upper-follicle stromal and checkpoint tiers. Antifibrotic and proangiogenic regimens that counter TGF-*β*–driven fibroblast activation and normalize matrix and vascular remodeling would be coupled with strategies that restore or functionally mimic checkpoint axes on the upper-follicle epithelium. Concomitant early mitigation of mechanical stressors could reduce upstream damage-associated molecular inputs that would otherwise stabilize the fibroblast-dominant circuit (36, 141, 142).

Beyond conventional biologics, the NEST framework serves as a conceptual scaffold for advanced therapeutic platforms supported by early proof-of-concept evidence, including cell-based approaches (e.g., Treg therapy) and acellular modalities (e.g., nanoparticle-mediated tolerance induction, gene-delivery systems, and extracellular vesicle–based therapeutics) (143–147). In doing so, NEST emerges as a unifying blueprint for multimodal therapeutic design in a new therapeutic era aimed not merely at suppressing inflammation, but at durably reconstructing follicular tolerance.
